# Spontaneous inflammation and necrosis of the falciform and round ligaments: a case report and review of the literature

**DOI:** 10.1186/s13256-019-2335-x

**Published:** 2020-01-23

**Authors:** Astha Bhatt, Emmanuel Robinson, Steven C. Cunningham

**Affiliations:** Department of Surgery, St. Agnes Hospital, 900 Caton Avenue, MB 207, Baltimore, MD 21229 USA

**Keywords:** Falciform ligament, Necrosis, Inflammation, Round ligament, Ligamentum teres, Abdominal pain, Gallbladder mimicry

## Abstract

**Background:**

Necrosis of the falciform and round ligaments is extremely rare, thus making the diagnosis challenging. It is often misdiagnosed as gallbladder pathology due to the presenting symptoms. Due to the rarity of this pathology, there is limited literature available.

**Case presentation:**

A 53-year-old white man presented to our hospital with signs and symptoms of gallbladder pain but turned out to have the rare entity of necrosis of the falciform and round ligaments. An extensive review of the world literature was performed using PubMed. Manual cross-referencing of reference lists was performed to obtain all available articles. The personal operative log of the senior author was also searched to reveal one additional case. Statistical analysis was descriptive only, given the small number of reported cases. Thirty-nine articles were found, among which forty-three case were identified, and one additional case was extracted from the operative log of the senior author. Unlike previous reports, we found that isolated inflammation and necrosis of the ligaments occurs at nearly the same frequency in both men and women, not predominantly in women as previously reported in smaller series. The mean age at presentation was 59.5 years old, and cases were typically initially diagnosed as gallbladder pathology, most commonly acute cholecystitis. Computed tomography more frequently than ultrasound revealed the falciform and round-ligament pathology.

**Conclusions:**

Isolated falciform and round-ligament inflammation and necrosis is a rare condition that is difficult to diagnose because it can present mimicking a wide variety of intra-abdominal pathologies, particularly gallbladder pathologies. It is often best treated by laparoscopic resection. Unlike prior reports, our review of the literature, which is the largest that we know of to date, shows that males and females are equally affected. Greater awareness of this entity will aid in future diagnosis.

## Introduction

The falciform ligament is a triangular, or falciform, fold of peritoneum stretching between the umbilical fissure of the liver and the anterior abdominal wall. The free edge of the falciform ligament is the round ligament (also called the *ligamentum teres*), which is a fibrous, cord-like remnant of the obliterated umbilical vein. It is a derivative of the embryonic ventral mesentery.

Unfortunately, due to its rarity, inflammation or necrosis of these ligaments is very difficult to diagnose preoperatively and is often confused with other more common causes of abdominal pain, such as gallbladder disease. The etiology of the inflammation and necrosis can be infectious or ischemic, in some cases associated with tumors, and often cryptogenic.

We present a new case and review the world literature on round and falciform ligamentitis to better characterize this rare condition. Due to the rarity of this condition and the limited literature available in the English language, we believe that this case and our multilingual literature review will allow medical practitioners to better establish this diagnosis and have an understanding of its clinical features.

## Case presentation

A 53-year-old white man presented to our hospital with a 3- to 4-day history of progressive epigastric and right upper quadrant pain. He had chills but no fever, nausea, or vomiting. His past medical history was significant for hypertension, borderline diabetes, chronic obstructive pulmonary disease, and sleep apnea. His past surgical history included umbilical hernia repair. His family history was significant for bladder and colorectal cancer. He smoked one pack of tobacco per day for many years but did not use alcohol or recreational drugs. He had no known environmental exposures, and he was currently unemployed. Upon admission, his blood pressure was 97/71 mmHg, heart rate was 67 beats/minute, temperature was 97.8 °F, and weight was 285 pounds.

A detailed physical examination did not demonstrate any abnormalities in the cardiovascular, pulmonary, neurological, and musculoskeletal systems but was significant for tenderness in the epigastrium and right upper quadrant of the abdomen, which was itself soft and rotund with obesity. The patient’s white blood cell count was 15,700/mm^3^. His hemoglobin, platelets, chemistries, and liver panel results were all normal, as was his urinalysis.

**Fig. 1:**
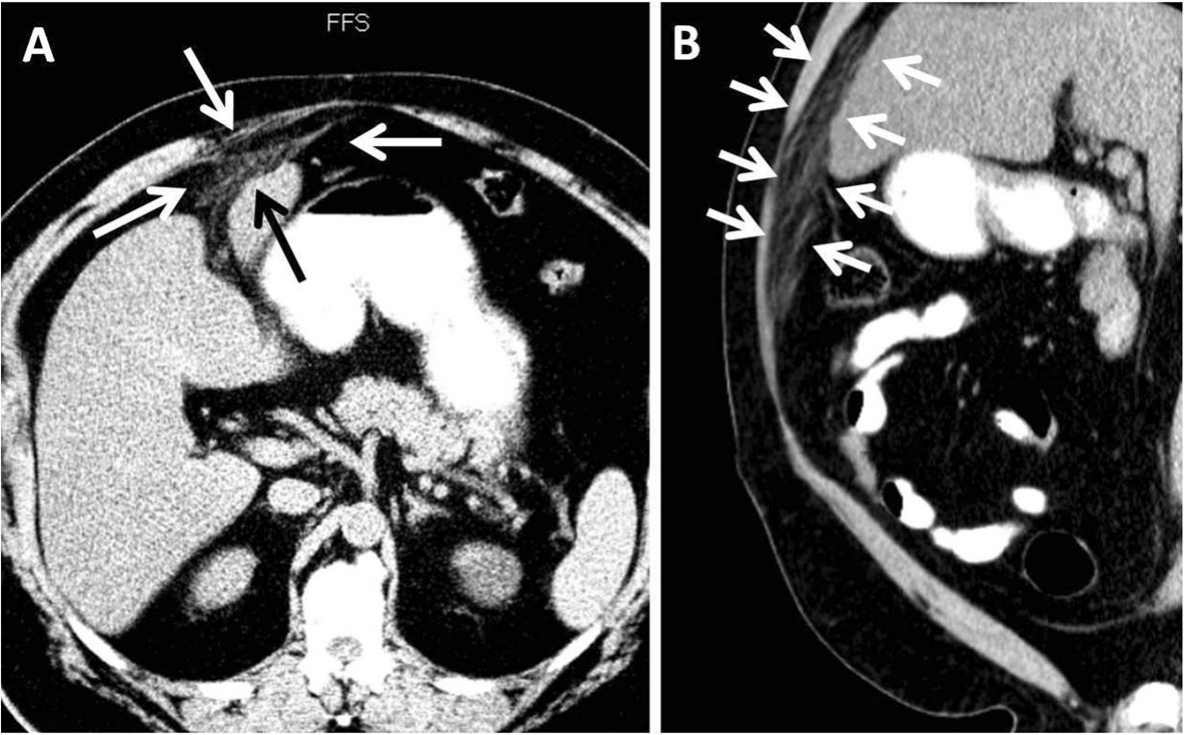
Axial (**a**) and sagittal (**b**) computed tomographic images showing fat stranding extending (arrows) from the umbilical fissure toward the umbilicus

Computed tomography (CT) showed fat stranding extending from the umbilical fissure, along the course of the falciform and round ligaments, anteriorly to the abdominal wall and inferiorly toward the umbilicus (Fig. 1). Ultrasound (US) showed cholelithiasis without signs of cholecystitis or biliary dilation. A hepatobiliary scan showed a low ejection fraction (2%) of the gallbladder and a patent common bile duct. Esophagogastroduodenoscopy (EGD) showed only mild erythema of the proximal body and greater curvature.

Upon admission, the patient was treated empirically with piperacillin and tazobactam 3.375 g intravenously every 6 hours, and in addition, he was given intravenous hydromorphone and ondansetron as needed for pain and nausea, respectively.

**Fig. 2:**
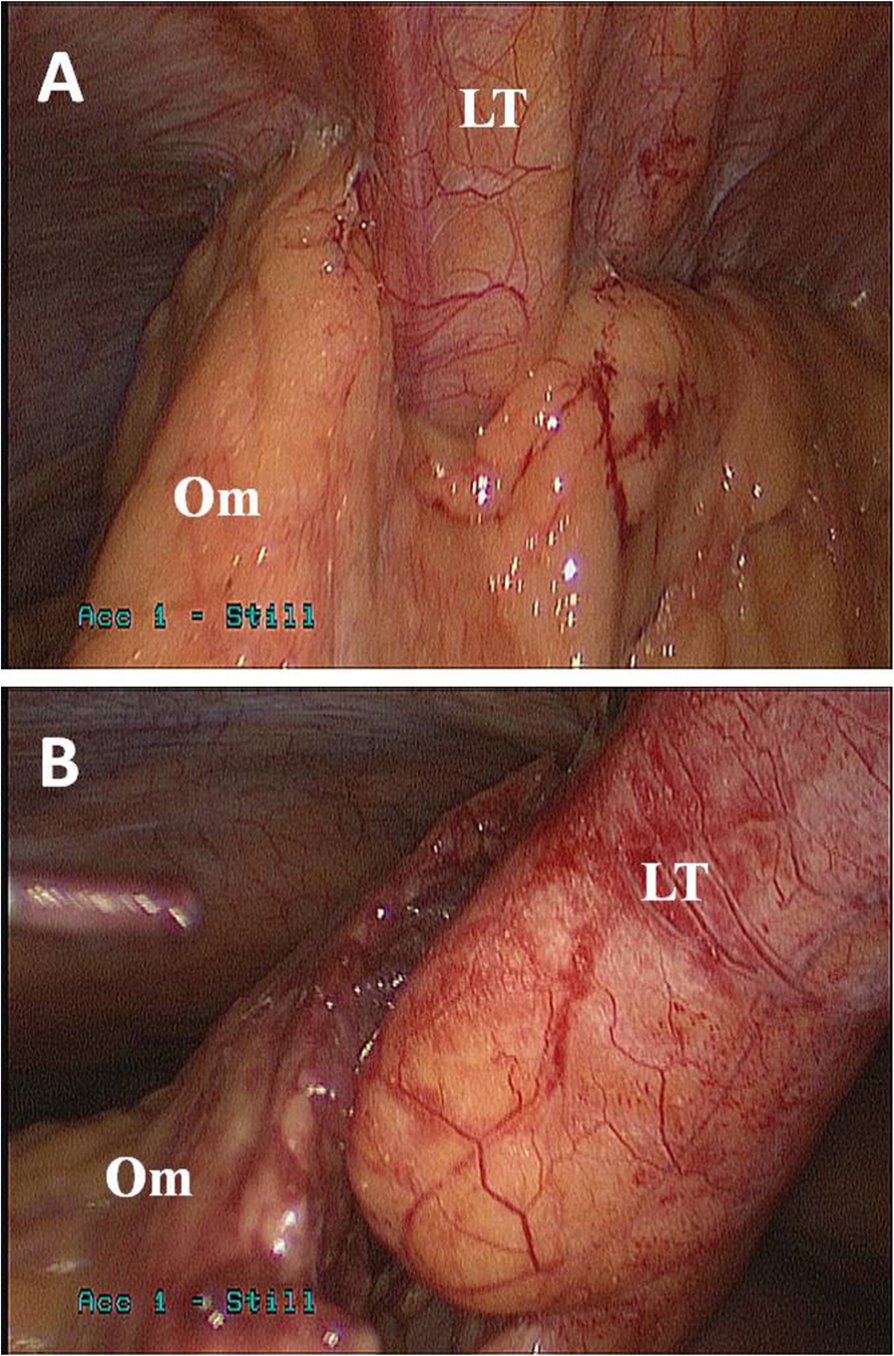
Intraoperative photograph of the omentum (Om) adherent to the ligamentum teres (LT) prior to (**a**) and during (**b**) dissection

Given his progressively worsening symptoms, he was taken for laparoscopic exploration. At operation, the falciform and round ligaments were severely inflamed and thickened, with adherent omentum (Fig. 2). The falciform and round ligaments and a small portion of involved omentum were resected. His gallbladder was distended but not inflamed and otherwise grossly normal. Given his low ejection fraction and gallstones, his gallbladder was removed to prevent future gallstone disease.

Pathologic examination revealed hemorrhagic fat necrosis and fibrin thrombi of the omentum, with hemorrhage and inflammatory changes of the falciform and round ligaments. The gallbladder pathology revealed only minor, microscopic evidence of chronic inflammation and cholesterolosis.

The patient’s postoperative outcome was good, and he was discharged on the second postoperative day. He failed to return for a postoperative follow-up visit, but 1 year later, he presented to the emergency room of our hospital with unrelated complaints but no abdominal pain.

## Discussion

We present a case of a 53-year-old man with a 4-day history of epigastric pain with associated abdominal tenderness. His laboratory values were significant for leukocytosis of 15,000/mm^3^. Imaging revealed fat stranding around the falciform ligament but no sign of acute cholecystitis. After an initial attempt at nonoperative management, the patient was brought to the operating room for diagnostic laparoscopy, given his persistent pain that was constant and located increasingly in the epigastrium. Laparoscopic exploration revealed isolated ligament necrosis of the falciform and round ligaments. This case is important to present for several reasons. First, it draws attention to the tendency of necrosis of these ligaments to mimic gallbladder disease. Second, it is unique in that the patient also was found to have a concomitant severely reduced gallbladder ejection fraction on a hepatobiliary scan. It is also noteworthy because, despite the availability of US and CT, this diagnosis of necrosis of the falciform and round ligaments was not recognized. Upon retrospective review of the patient’s CT scan, however, falciform ligament inflammation was appreciated, highlighting an important learning opportunity. One reason it was not fully appreciated initially was a lack of awareness of this entity, due both to its rarity and to the paucity of publications on spontaneous necrosis of the falciform and round ligaments. Finally, another important point highlighted by this case is that the treatment can be simple and effective via laparoscopic resection of falciform ligament.

In addition to reporting this case, we performed a literature search in PubMed (National Library of Medicine, National Institutes of Health) using keywords “falciform ligament necrosis,” “inflammation,” “round ligament,” and “ligamentum teres.” This review revealed further important findings, including that this entity occurs at nearly at the same frequency in both men and women, not predominantly in women as previously reported in smaller series.

Titles, abstracts, and full-text publications were obtained and screened for presentation of case reports of round or falciform ligamentitis. No language restrictions were applied. Full-text versions of all foreign articles were obtained. The following data were extracted from the selected articles: demographics, medical and surgical history, surgical technique used, and pathology findings. Screening of non-English papers was performed using standard web-based programs, with translation and confirmation performed by colleagues fluent in those languages. Data were summarized descriptively.

This literature search yielded 43 case reports in 39 publications, with an additional 2 cases derived from a manual search of references, but these were excluded because they were pediatric cases, and 1 additional case was found in the senior author’s operative log, for a total of 44 cases after excluding pediatric cases. The included reports were published between 1976 and 2014. The articles retained for this review were published in English (25 articles), French (6 articles), Danish (1 article), German (2 article), Japanese (1 article), and Russian (4 articles).

According to this literature review (Table 1), round and falciform ligament inflammation occurs slightly more frequently in males than in females but approaches a 1:1 ratio. Among the 43 published cases found during the review of literature, 21 of the patients were male, and 18 were female, with 4 cases in which the patient’s sex was not disclosed. In the reported cases, the average age of presentation was 59.5 years (excluding pediatric cases). The most common age group was 60–69 years in 10 of the reported cases, followed by the 70–79-year-old age group with 7 cases reported.

**Table 1:** Important findings from the literature review, including mean age at presentation, preoperative diagnostic findings, and postoperative pathology findings

Mean age at presentation	59.5 years old
Sex	21 males 18 females not disclosed in 4 cases
Preoperative diagnostic findings	Cholecystitis/choledocholithiasis 34.3% Acute pancreatitis 8.6% Intra-abdominal abscess 17.1%
Open vs. laparoscopic	Open 57.9% Laparoscopic 23.7% Medical/conservative management 18.4%
Pathology findings	Isolated pathology 73.7% Cancer or tumor 10.5% Herniation 10.5% Combined pathology 26.3%

Among the reviewed cases, four patients had a past medical history of hypertension (including our patient), five patients had diabetes mellitus (also including our patient), two patients had previous choledocholithiasis, and one had cholangitis due to periampullary carcinoma. There was no mention of coronary artery disease, except in one patient. There was no clear correlation between any medical condition or prior surgery and incidence of falciform ligament inflammation.

The most common presenting symptom was right upper quadrant and epigastric pain, as well as fever, and the most common physical examination finding was tenderness in the epigastrium and the right upper quadrant, with peritoneal signs being reported in three cases. Laboratory values were reported in only four articles, and inconsistently, precluding any meaningful analysis of laboratory values.

Regarding diagnosis, most patients received their diagnosis intraoperatively. The vast majority underwent US of the right upper quadrant and CT. The most common presumed etiology for this presentation was gallbladder disease, with 12 such cases of gallbladder mimicry: 7 were cases of suspected acute cholecystitis, 4 cases were cholelithiasis, and 1 was a probable acalculous cholecystitis. Other common preoperative differential diagnoses were acute pancreatitis and diffuse peritonitis secondary to perforated duodenal ulcers. A rarer etiology was small-bowel obstruction secondary to epigastric herniation. Only one of all the reviewed cases had round-ligament necrosis as a primary diagnosis [1].

US imaging typically revealed hypoechoic infiltration of the round ligament or infarction of the fatty appendage of the ligamentum teres suggestive of acute appendagitis of the ligament. Moreover, few cases of hepatic and biliary lesions and masses captured by US were reported [2, 3]. CT was more commonly used and likely more accurate in evaluating hepatic and falciform ligament lesions. In the literature, preoperative CT findings were confirmed intraoperatively. These findings included falciform ligament abscess [4], desmoid tumor appended to the round ligament [5], and infiltration of the round ligament [6].

Regarding treatment, in a majority of the cases, surgeons opted for an open surgical approach over a laparoscopic approach; such an option was presumably available for a majority of the cases, because only eight articles were published before 1990. Among the reviewed cases, 22 patients underwent laparotomy; of these, 9 had laparoscopic procedures, and 7 were treated with medical management only, giving a ratio of 2.5:1. However, this ratio decreased for more recent cases (over the past decade); among cases published after 2009, there were 7 cases of open procedures and 5 laparoscopic surgeries (including our patient’s case), giving a ratio of 1.4:1. Cholecystectomy was performed in addition to the partial or total resection of the falciform ligament in eight (18%) of the analyzed cases.

The most common intraoperative finding was partial or total necrosis or gangrene of the round ligament with abscess, which was reported in 24 (55%) cases, and in a majority of cases, this was confirmed by postoperative pathology. Involvement of the gallbladder in the inflammatory process was noted in four cases (10%). There was only one mention in the analyzed literature of involvement of the liver [1], but none required any resection of the liver segments. On four occasions, herniation of the small bowel through the ligament was found without any inflammatory process [7–10]. Tumors of the falciform ligament have rarely been reported. In some cases, tumor from surrounding structures such as invasion of the round ligament by lieberkuhnian adenocarcinoma [11], lymphangioma of the falciform ligament [3], and abdominal desmoid tumor encapsulating the round ligament have also been reported [5].

Inflammation of the falciform ligament can be explained by infection of surrounding structures such as the gallbladder, liver, peritoneum, and thoracic or abdominal wall. Isolated inflammation of the falciform ligament has also been reported. In addition, torsion of preexisting cysts in the falciform ligament can lead to infarction and gangrene [12, 13]. Engorged vessels of the collateral circulation between portal and azygous systems in cases of portal hypertension may compress the falciform and round ligaments [14]. Congenital anomalies such as high insertion, partial defects, or deviation have also been reported [15, 16].

Most commonly, as in our patient’s case, the falciform ligament and the round ligament are simultaneously inflamed. This may be attributed to the arterial and lymphatic supply to the ligaments. The right hepatic artery gives off a branch, which enters the round ligaments and gives off two to three branches that anastomose with tributaries from the superficial inferior epigastric artery. The venous flow is through the paraumbilical veins. The superior veins drain the medial diaphragm and traverse the upper and lower veins to communicate with tributaries of anterior abdominal veins, mainly epigastric vessels [17]. Lymphatic flow is from the retroperitoneum through the lesser omentum [18]. This communication explains how septic or embolic sources in the liver, retroperitoneum, and abdominal wall may cause falciform ligament infection, gangrene, or necrosis [19].

Because this entity is rare, the choice of surgical intervention is not clear. Historically, laparotomy was performed. Now, with advances of laparoscopic surgery, it is the safer and easier method for complete or partial excision of the falciform ligament. It also depends on the severity of the patient’s condition, the extent of disease, and the surgeon’s expertise. The operation performed in our patient was a subtotal resection of the falciform ligament, leaving only a small part of viable ligament in the umbilical fissure between segments III and IV. There has been only one report of a case in which a complete resection of the falciform ligament has been performed [20]. Although our patient had both cholesterolosis of the gallbladder and a low ejection fraction, these findings have been shown in a large study of nearly 7000 cholecystectomies not to correlate with each other [21].

## Conclusions

Inflammation and necrosis of the falciform and round ligaments is a very uncommon entity with limited literature available. However, some pearls can be gleaned from our case report and literature review. The mean age of presentation is 60 years old, and women and men are equally affected. Patients typically present with symptoms mimicking gallbladder disease, such as acute cholecystitis or symptomatic cholelithiasis. In cases of right upper quadrant or epigastric tenderness or peritonitis without positive findings of gallbladder inflammation or stones, or free fluid or gas, surgeons should be more suspicious of alternative pathology, such as round/falciform ligamentitis. CT is preferred to US to make the diagnosis. Surgical management is the definitive treatment, and a minimally invasive approach is preferred when feasible.

## Abbreviations

CT: Computed tomography
US: Ultrasound
